# Deep learning and wing interferential patterns identify *Anopheles* species and discriminate amongst Gambiae complex species

**DOI:** 10.1038/s41598-023-41114-4

**Published:** 2023-08-25

**Authors:** Arnaud Cannet, Camille Simon-Chane, Mohammad Akhoundi, Aymeric Histace, Olivier Romain, Marc Souchaud, Pierre Jacob, Darian Sereno, Karine Mouline, Christian Barnabe, Frédéric Lardeux, Philippe Boussès, Denis Sereno

**Affiliations:** 1 Direction des Affaires Sanitaires et Sociales de la Nouvelle-Calédonie, Nouméa, France; 2 ETIS UMR 8051, ENSEA, CNRS, Cergy Paris University, 95000 Cergy, France; 3 Parasitology-Mycology, Hopital Avicenne, AP-HP, Bobigny, France; 4 CNRS, Bordeaux INP, LaBRI, UMR 5800, Univ. Bordeaux, 33400 Talence, France; 5 InterTryp, IRD-CIRAD, Infectiology, Medical entomology & One Health research group, Univ Montpellier, Montpellier, France; 6 MIVEGEC, CNRS, IRD, Univ Montpellier, Montpellier, France

**Keywords:** Diseases, Zoology, Entomology, Biodiversity

## Abstract

We present a new and innovative identification method based on deep learning of the wing interferential patterns carried by mosquitoes of the *Anopheles* genus to classify and assign 20 *Anopheles* species, including 13 malaria vectors. We provide additional evidence that this approach can identify *Anopheles* spp. with an accuracy of up to 100% for ten out of 20 species. Although, this accuracy was moderate (> 65%) or weak (50%) for three and seven species. The accuracy of the process to discriminate cryptic or sibling species is also assessed on three species belonging to the Gambiae complex. Strikingly, *An. gambiae, An. arabiensis* and *An. coluzzii,* morphologically indistinguishable species belonging to the Gambiae complex, were distinguished with 100%, 100%, and 88% accuracy respectively. Therefore, this tool would help entomological surveys of malaria vectors and vector control implementation. In the future, we anticipate our method can be applied to other arthropod vector-borne diseases.

## Introduction

Pathogens transmitted by arthropods are devastating infectious agents and scourge the human population worldwide. Currently, the 3.719 valid species of Culicidae are classified into 2 subfamilies, Culicinae and Anophelinae (https://mosquito-taxonomic-inventory.myspecies.info/valid-species-list/ accessed on 8 Aug., 2023). Among Culicidae insects belonging to the *Anopheles* Meigen, 1818, some are proven vectors of protozoan pathogens (*Plasmodium* sp*.*)^1^, viruses (Zika, Rift Valley fever, etc.)^2,3^, bacteria (*Rickettsia felis*)^4,5^ pathogens, or a limited number of species are thought to act as phoretic vector for *Dermatobia hominis*^6,7^. Some species (*An. gambiae* Gilles, 1902, and *An. bancrofti* Gilles, 1902) are proven vectors of a filarial pathogen, *Wuchereria bancrofti*^8^. Nevertheless, pathogens’ detection in field-caught insects doesn’t imply their vectorial importance, and in all cases, additional demonstrations, including experimental infection and transmission, are needed to firmly prove their vectorial status^2^. Overall, malaria is a life-threatening disease with over 600 000 deaths annually (World Malaria Report; https://www.who.int/teams/global-malaria-programme/reports/world-malaria-report-2022), and *Anopheles* species as the vector are of significant importance in public health and disease control.

The *Anopheles* genus encompasses eight subgenera, with about 500 valid named species and 49 subspecies (https://www.itis.gov/ accessed on the 28th of November 2022 returns 477 named species). Out of 8 described subgenera, 4 hold named species whose medical or veterinary interests are documented (*Anopheles* Meigen, 1818; *Cellia* Theobald, 1905; *Kerteszia* Theobald, 1903; *Nyssorhynchus* Blanchard, 1902). The latter is exclusively present in the new world (south, central, north America, and the Caribbean). The highest species richness is recorded in the Asian and African continents, followed by the American, Oceanian, and European continents. Roughly 140 species have a well-documented medical or veterinary interest (Data compiled from WRBU website, https://wrbu.si.edu/ accessed on the 28th of November 2022).

Complexes of species encompass closely related organisms that are so similar in appearance and other features that the understanding of boundaries between them needs to be clarified. The study of differences between individual species of the complex requires the identification of minute morphological details, tests of reproductive isolation, or DNA-based methods, such as molecular phylogenetics and DNA barcoding. At least 27 species complexes, with up to 10 species per complexes, are documented for the *Anopheles* genus. The “Gambiae complex” (*Anopheles gambiae *sensu lato) was recognized in the 1960s^9^. Since then, additional evidence on population subgroups and genetic diversity in this African malaria vector emerged^10–12^. To date, the Gambiae complex gathers nine closely related species (*An. amharicus* Hunt, Wilkerson & Coetzee, 2013; *An. arabiensis* Patton, 1905; *An. bwambae* White, 1985; *An. coluzzii* Coetzee & Wilkerson, 2013; *An. gambiae* Giles, 1902; *An. melas* Theobald, 1903; *An. merus* Dönitz, 1902; *An. quadriannulatus* Theobald, 1911), and a last included species, *An. fontenillei* Barron*,* 2019, collected in Gabon^13^, of which seven are proven vectors of various *Plasmodium* sp. Some species (*An. melas, An. merus, An. quadriannulatus, An. amharicus,* and *An. bwambae*) do not overlap in their distributions, unlike 3 of the most important malaria vectors in sub-Saharan Africa: *An. gambiae s. s., An. coluzzii* and *An. arabiensis*. Entomological surveys and follow-up of malaria vectors in time and space require identification at the species level because malaria is transmitted by multiple and often barely morphologically distinguishable mosquito species that differ in their longevity, behaviors, and vectorial competence and hence vectorial capacity^11,14^.

Entomological investigations are fastidious and nowadays primarily dependent on highly skilled specialists. Morphology-independent methodologies, even the most complex ones as geometric morphology, rely on genetic, protein, or other specific biochemical markers (DNA barcode, MALDI-TOF, Near, and Middle-infrared spectroscopy) can partially resolve the taxonomic status of some specimens but cannot be considered as amenable for entomological survey^15–19^. In addition, acoustic, like flight tone and wing beat^20–22^, and optical characters like WIPs (Wing Interference Patterns)^23–27^, were developed and tested on insects’ members of various families. Finally, the advances in Deep learning (DL) processes, a branch of machine learning (ML) and artificial intelligence (AI), have incredibly increased the identification capability of arthropods^27–34^.

In this study, we aimed to develop a supervised DL approach on *Anopheles* WIPs to predict individual species. We tested the robustness of this approach in differentiating closely related species belonging to the same “Gambiae complex,” *i.e., An. gambiae*, *An. coluzzii* and *An. arabiensis*, using insectary-reared specimens. The results prove how this low-cost, artificial intelligence-based approach can determine the species composition of natural vector populations and constitute a new identification tool in the fight against malaria.

## Material and methods

### Anopheles collection and storage

The first WIPs reference collection of Culicidae gathers samples belonging to the Anopheles genus using well-established laboratory breeds of *An. gambiae*, *An. coluzzii* and *An. arabiensis* and *An. stephensi* (MIVEGEC, IRD Montpellier, France and IRRS Bobo Dioulasso, Burkina Fasso). Specimens were also selected in the ARIM collection (https://arim.ird.fr/) of IRD (Institut de Recherche pour le Développement). In addition, specimens collected *in natura,* whose identification was performed at the time of their traping with available regional morphological identification keys, and confirmed before their entry in the ARIM collection, were also included in the database. The description of the samples used in this study is given in Table 1.

**Table 1:** List of *Anopheles* species and description of samples included in the dataset.

*Anopheles* spp*.* in the database	Medical interest^$^	Origin	Year	N	Country code^&^	Identification performed by
*Anopheles* Meigen, 1818						
*An. maculipennis*	Yes	W	1960–2010	14	250, ND	Le Goff, others
*An. obscurus*	No	W	1957–1962–1988	24	178, 120 ND	Adam, Mouchet, others
*An. paludis*	No	W	1959, 1968, 1988	20	120, 854	Hamon, others
*An. punctimacula*	Yes	W	ND	11	604	Villanueva
*Cellia *Theobald, 1902						
An. arabiensis*	Yes	C	2015	43	854	Mouline, Lefèvre
*An. barberellus*	No	W	1956	10	384	Adam
*An. cinctus*	No	W	1958	14	384	Hamon
*An. cinereus*	Yes	W	1959	19	504	Bailly-Choumara
An. coluzzii*	Yes	C	2015	127	854	Mouline, Lefèvre
*An. demeilloni*	Yes	W	1959	11	178	Hamon
*An. funestus*	Yes	W	1998	197	450	LeGoff
An. gambiae*	Yes	C	2014	41	250, 638	Boussès, Noel
*An. listeri*	No	W	1966, 2010	13	34	LeGoff, Gilot
*An. machardyi*	No	W	ND	10	ND	Gilot
*An. mascarensis*	Yes	W	2010	60	450	LeGoff
An. nili**	Yes	W	1959, 1966, 1967	12	120, 854	Hamon
*An. pharoensis*	Yes	W	1957, 1995	20	562	Bruhnes, Adam
*An. squamosus*	No	W	1951, 1952, 1991, 1995	20	120, 466, 854	Holstein, Bruhnes, Hamon
*An. multicolor*	Yes	W	ND	10	504	Bailly-Choumara
*Nyssorhynchus* Blanchard, 1902						
*An. darlingi*	Yes	W	2003	34	68	Lardeux
Subtotal				710		
*Anopheles spp with documented WIPs that had not undergone the DL process for species recognition*						
*Anopheles* Meigen, 1818						
*An. apimacula*	No	W	ND	6	558	Grimaldo
*An. atroparvus*	Yes	W	1966, 2012	4	250	Boussès, others
An. claviger***	Yes	W	1966	6	686	Gilot
*An. labranchiae*	No	W	1972	8	504	Bailly-Choumara
*An. pseudopunctipennis*	Yes	W	ND	8	ND	Villanueva
*An. ziemani*	Yes	W	1967	6	504	Bailly-Choumara
*Cellia *(Theobald, 1902)						
*An. brohieri*	No	W	1965	3	384	Brunhes
An. carnevalei**	Yes	W	ND	2	ND	ND
*An. dthali*	No	W	1962	3	262	Mouchet
*An. dureni*	No	W	1955	9	854	Hamon
*An. flavicosta*	No	W	1966	6	854	Brunhes
*An. hargreavesi*	No	W	1957	7	120	Adam
An. marshallii****	No	W	1965	5	180	Hamon
An. melas*	Yes	W	1964, 1996	9	666	Rodhain, Faye
*An. moucheti*	Yes	W	1991	7	120	Mouchet
*An. pretoriensis*	No	W	1958	7	854	Adam
*An. rhodesiensis*	No	W	1967	4	854	Hamon
*An. rufipes*	Yes	W	1959	7	854	Adam
*An. sergenti*	Yes	W	ND	8	504	Bailly-Choumara
*An. stephensi*	Yes	C	ND	5	250	ND
*Nyssorhynchus* Blanchard, 1902						
*An. aquasalis*	Yes	W	ND	6	ND	ND
*An. albimanus*	Yes	W	1964	3	1964	Rodhain
*An. braziliensis*	Yes	W	N	4	ND	ND
Subtotal				133		
Total				843		

*Gambiae complex.

**Nili complex.

***Claviger complex.

****Marshallii complex.

^$^Medical interest according to the WRBU database (https://wrbu.si.edu/vectorspecies?field_family_target_id=1194&title=&field_mt_products_tags_target_id=&field_pathogens_target_id=&field_geographic_locations_target_id=&items_per_page=30) and Wilkerson et al.^35^.

^&^ISO 3166-1 country code available at (https://www.atlas-monde.net/codes-iso/).

0rigin: the sample's origin, W, wild; C, colony; N, number of picture in the database.

### Image acquisition and database construction

The same standard operational procedures (SOP) described to capture WIPs of *Glossina* were also used for *Anopheles*^27^. This process is easy to handle and inexpensive. It consists of dissecting the wings and mounting them on a glass slide. A cover slide was deposited, annotated specimens were photographed using the xVH-Z20r camera, and the VH K20 adapter (Keyence™) was set to 10°of illumination incidence. The function High Dynamic Range (HDR) was used for all pictures. All pictures were enlarged to get sized photos that exclude the wing size as a discriminating criterion for species identification by deep learning approaches. Geographical origin, sampling date, and the sex and identity of the filed-caught species and the entomologist who identified them in the sampling location were recorded individually. The numerical parameters of the camera were as follows: white Balance 3200 K, Shutter Speed 1/15(sec), gain 0db, frame rate 15F/s, brightness 15%, texture 15%, contrast 45%, color 100%. The luminosity, contrast, shadow, reflection, and saturation were settled at 80, 100, 0, 0, and 100% using Windows 7 familial edition. All pictures were dusted off manually before being filled in the database.

### Collected dataset, image pre-processing, and dataset splitting for training/learning and validation

The annotated image dataset, including 843 pictures of 42 Anopheles species belonging to 3 subgenera (*Anopheles*, *Cellia*, *Nyssorhynchus*) were prepared to undergo learning *Anopheles* classification. For training purposes, the sample sex, geographic origin (population), age, and physiological state (blood feed or not) were not considered to get a general classifier model. The 4688 pictures of WIPs belonging to the Diptera family encompassing Glossinidae, Psychodidae, Culididae, and other genera were added. Under-sampled *Anopheles* species (less than ten samples/pictures) and *An. multicolor* were discarded for the training of *Anopheles* identification at the species level to prevent overfitting. Still, they were included in the training dataset at the subgenus level. All processed images were resized to 256 and 116 pixels for width and height, respectively. Pixel values were normalized within the (0,1) range. The dataset was then prepared for k-fold cross-validation, with k = 5, similar to what have been performed for *Glossina sp* WIPs analyses^27^. K-fold cross-validation is a classic approach to evaluate the robustness of a machine learning method, including Deep Learning ones. For this study, the dataset was randomly shuffled and partitioned into k equal-size subsets with similar class distributions. A separately learned classifier was evaluated for each subgroup using the kth of all datasets for validation and the remaining k−1 as training data.

**Figure 1:**
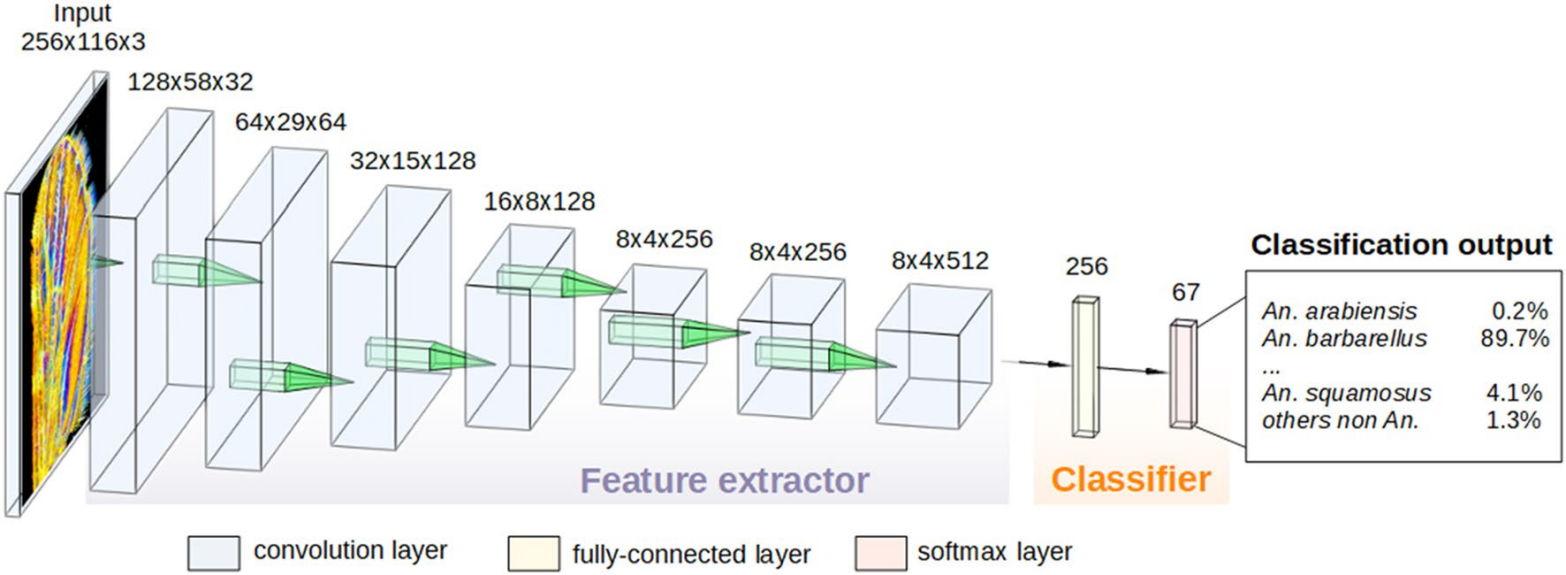
Schematic representation of the pipeline process developed for *Anopheles* identification using the Convolutional Neural Network approach. Example of classification output with the associated probability. The class of a given *Anopheles* WIPs image is predicted by two steps: (1) extracting hierarchical features (Convolutional layer) and (2) classifying these features (Fully-connected layer and softmax layer). In the feature extractor part, feature maps generated by filters at each convolution layer are indicated. These feature maps are used for visualization by weighting them with channel-wise averaged gradients.

This strategy allowed measuring the mean accuracy of the five distinct generated classifiers. Among all existing machine learning methods, Deep Convolutional Neural Networks and their different architectures have shown in the last decade to be the most adapted for image classification. Compared to classic shallow methods (Support Vector Machine, Random Forest, and Boosting-based approaches for the main ones), they do not need hand-crafted features as input of the learning process: the selection of the best features is intrinsic to the method itself and is particularly well adapted to the particular scenario of WIPs. A pipeline overview of the complete training procedure using CNN is shown in Fig. 1.

### Training of the convolutional neural network (CNN)

The original CNN architecture MobileNet^36^, ResNet^37^, and YOLOv2^38^ architecture were deemed for the automatic classification with the abovementioned dataset. Compared to classic Deep Learning, ours is more compact to cope with our dataset's specificity in terms of size; therefore, thinner image recognition and classification architecture were developed to consider its reduced size. The first one is inspired by MobileNet, which takes advantage of depth-wise convolution^36^. We propose to work with only one depth-wise convolution per layer of the CNN architecture to reduce the complexity and the number of extracted features. In addition, batch normalization was set to speed up and stabilize the training process^39^.

In this first compact CNN architecture based on MobileNet, two interconnected layers like VGG^40^ for YOLOv2 were applied with a DarkNet-19^38^architecture. As this kind of architecture tends to over-fit the training set (which means a lack of generalization of the performance when other data than the training data set is considered), we tested two reduced architectures, i.e., using 1 or 2 scales less than the original network. For clarity, we called them DarkNet-9 (8 convolution layers and one classification layer) and DarkNet-14 (13 convolution layers and one classification layer). We also reproduced the ResNet18 architecture^37^ and trained it from random initialization. Even if this architecture seems too “deep” (may lead to overfitting) compared to our other architectures, the intrinsic properties of ResNet18, residual connections, allow convergence of the training procedure. Finally, a standard approach (shallow approach) based on extracting SURF descriptors (an efficient implementation of the classic SIFT descriptors), a Bag of Features (BoF) representation using a 4000 codewords dictionary, and an SVM with a standard polynomial kernel similar to it was proposed in Sereno et al.^26^. For each task, we only use 1 fully connected layer with the softmax activation to predict the probability that an image belongs to the correct class. We train our networks using Stochastic Gradient Descent (SGD) with a learning rate of 10^2^ and a momentum of 0:9 for 30 epochs. The method was developed on a workstation with a quad-core CPU at 3.0 GHz and 16Go RAM. Information on the training options, accuracy and sensitivity, as well as the code, are available at https://github.com/marcensea/diptera-wips/commit/12f39ab500a3f820cfb817c67ef25c580942301d.

From the appurtenance probabilities matrix, an Euclidean distances (function *dist* in R package *stats*) distance matrix between pictures was computed. Then, from this distance matrix, a hierarchical cluster representation showing all photos of the test dataset was drawn using the average method of clustering (function *hclust* in R package *stats*) and plotted using the *ape* functions of the R package.

## Results

### Wing interferential pattern according to anopheles wings genera, species, sex, and date of sampling

#### Sexual dimorphism

**Figure 2:**
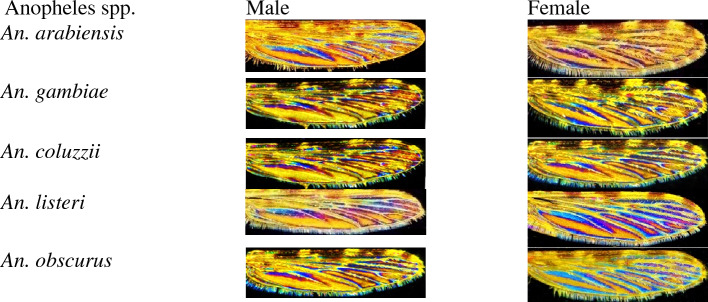
Wing interferential Patterns for male and female specimens of some *Anopheles* species.

**Figure 3:**
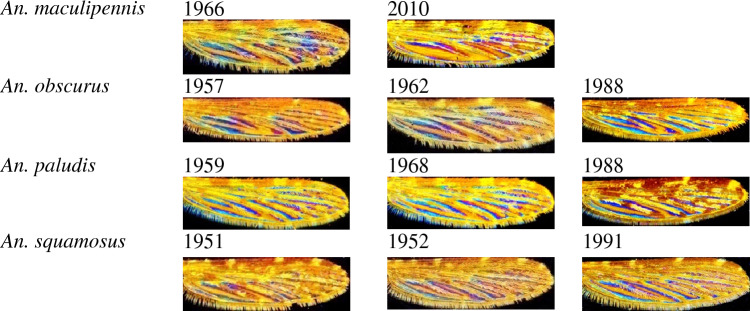
Wing Interferential Patterns of *Anopheles* specimens collected at various periods.

WIPs significantly vary among specimens belonging to different species but moderately amongst specimens of the same species or between sexes (Fig. 3). WIPs were explored on the broader panel of *Anopheles* specimens available and from 3 subgenera over the eight currently described. We previously documented the conservation of the interferential pattern on the wings of *Glossina* according to the position of the radial symmetry (intrados/extrado) and axial symmetry (left and right)^27^. We also investigate the sexual dimorphism of WIPS in studied samples (Fig. 2). Sexual dimorphism of WIPs is documented for numerous dipteran families, including Culicidae, Glossinidae, Muscidae, Calliphoridae, Ceratopogonidae…^41–43^. Picture of WIPs disclosed that for the Anopheles specimens we examined, the sexual dimorphism is weak and difficult to delineate with the naked eye (Fig. 3). A more in-depth study would be necessary to investigate its presence.

#### Date of sampling

Knowing that the sample dataset is filled with a variety of specimens collected and identified as early as 1951, we checked the stability of WIPs according to the age of collection of the specimen (Fig. 3). WIPs, when microscopically observable, appear unaffected by the conservatory period, allowing us to enrich our dataset with samples from the IRD collection. Although in some older samples and/or heavily damaged ones, WIPs cannot always be revealed. This lack of WIPs happened for 50% of specimens collected before the 80 s and preserved in the collection (Data not shown).

### Training and classification

We explored the training classifier accuracy on the *Anopheles* dataset and on datasets of Culicidae that do not belong to the *Anopheles* genus (non-Anopheles*)* and from mosquitoes that do not belong to the Culicidae family (non-Culicidae), as negative samples. We trained the CNN on such a combination to improve the model's accuracy. The database initially filled with a total of 843 pictures of *Anopheles* sp. WIPs pictures, 710 illustrating species documented with more than ten pictures and 133 with less than 10. Our dataset contains photos of species acting as primary vectors of viruses, parasites, or bacteria having a medical interest (Table 1). Our database is filled with 25 *Anopheles* sp, out of the 140 with documented medical or veterinary interests. Overall, the WIPs of 43 *Anopheles* species were filled in our dataset. However, only 20 species have encompassed the training process because at least ten pictures are available in our dataset. The other specimens were used only to train the classifier recognition at the genus (*Anopheles*) and subgenus (*Anopheles*, *Cellia*, *Nyssorhynchus*) taxonomic levels.

#### Classification at the genus level

Using this dataset, we first ascertained the accuracy of the process to discriminate the *Anopheles* genus Meigen, 1818, from other members of the Culicidae Meigen, 1818 family and belonging to the Culicinae Meigen, 1818 subfamily. These specimens belonged to the *Culex* Linnaeus, 1758; *Lutzia* Theobald, 1903; *Aedes* Meigen, 1818. Non-Culicidae sample members belonging to the Psychodidae, Glossinidae, and Ceratopogonidae families were also filled in the dataset to test the classification accuracy. The automatic classification process accuracy for *Anopheles* Meigen, 1818 is incredibly high, with more than 99% of accuracy (Table 2). A sole picture was badly classified as belonging to the *Culex* genus.

**Table 2:** Accuracy tests of the DL (Deep Learning) process for the *Anopheles* (Meigen, 1818) genus assignation. Accuracy values are in bold.

	Predicted		
	*Anopheles*	other genera	N
Truth			
*Anopheles* N (**Ac%**)	139 (**99.3%)**	1	140
Other genera N (**Ac%**)	1	878 (**99.9%**)	879

*Ac* accuracy, *N* number of pictures.

#### Classification at the subgenus level

In the second step, we investigate the capacity of our DL process to correctly address the identification of the specimens at the subgenus level. The training and testing dataset included a set of 833 pictures representative of 43 Anopheles species and three subgenera. Table 3 shows that the subgenus assignation accuracy is high for *Cellia*, moderate for *Anopheles*, and faint for *Nyssorhynchus*. The *Cellia* subgenus is documented by more species and pictures, followed by the *Anopheles *subgenus and the *Nyssorhynchus*. Therefore, the accuracy discrepancy ranging from 38.8% to 96.6% might be due to the low representativity of species and specimens for the *Anopheles* and *Cellia* subgenera. In addition, the selected descriptors and the training process might need to be revised to train an accurate classifier to identify *Anopheles* at the subgenus taxonomic level. These questions must be further addressed.

**Table 3:** Accuracy tests of the DL process at the subgenus level. Accuracy values are in bold.

Subgenera	Predicted			
	*Anopheles*	*Cellia*	*Nyssorhynchus*	N
Truth				
*Anopheles* N (**Ac%**)	28** (54.9%**)	15 (**29.4%**)	8 (**15.6%**)	51
*Cellia* N (**Ac%**)	5 (**2.4%**)	204 (**96.6%**)	0 (**0.0%**)	209
*Nyssorhynchus* N (**Ac%**)	4 (**22.2%**)	7 (**38.8%**)	7 (**38.8%**)	18
			Total	278

*Ac* accuracy, *N* number of pictures.

#### Classification at the species level

**Figure 4:**
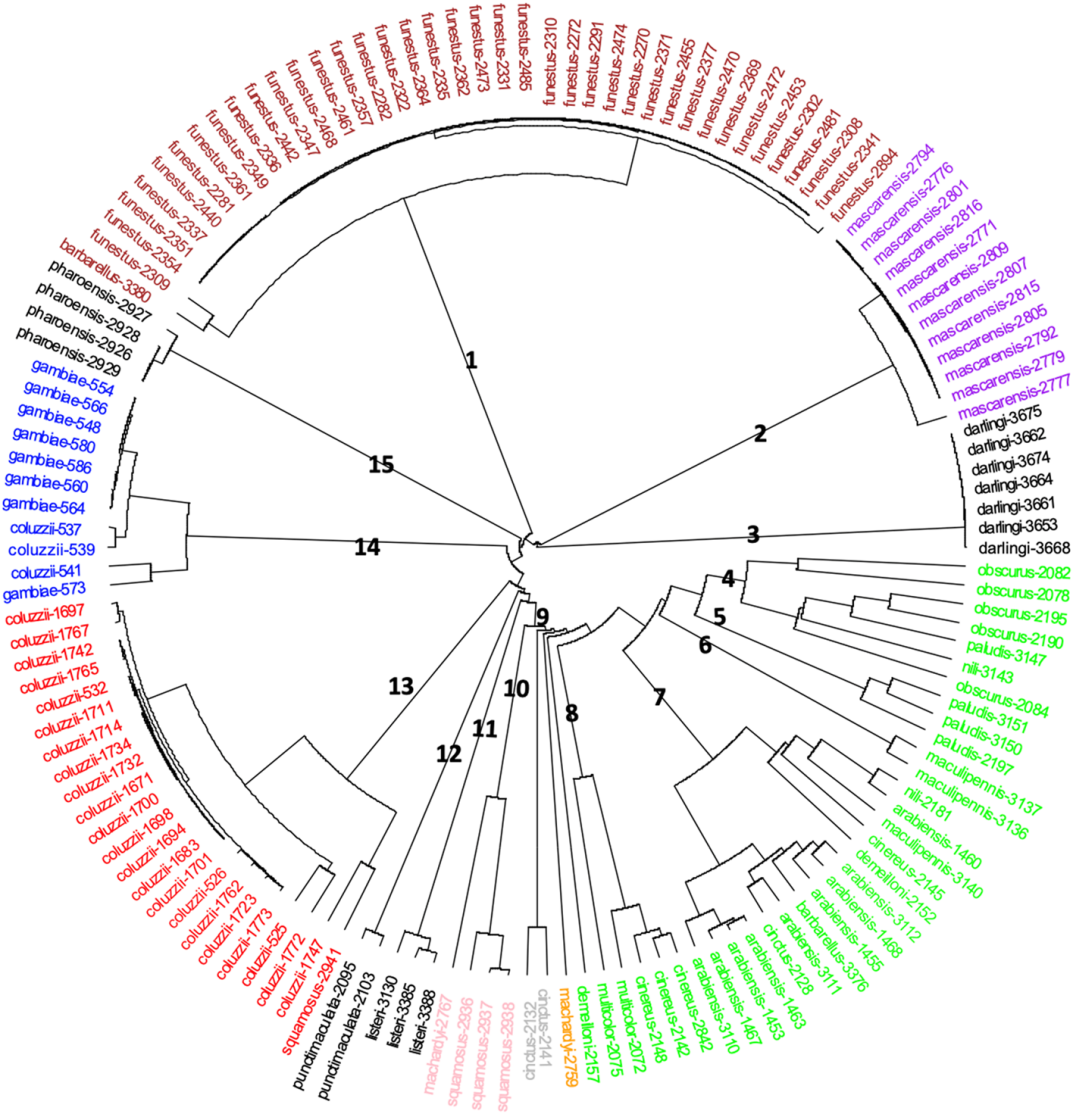
Circular cluster analysis representation of the *Anopheles* test dataset.

A circular dendrogram reflecting the proximity of each picture belonging to the *Anopheles* dataset was drawn (Fig. 4), depicting the presence of clusters. Some clusters match all the pictures of WIPs of the same species: *An. mascarensis,* cluster 2; *An. darlingi* cluster 3; *An. listeri*, cluster 11; *An. punctimacula*, cluster 12; *An. pharoensis,* cluster 15. Other clusters include all members of the same species plus some pictures of other related species: cluster 1, all *An. funestus* pictures plus one picture of *An. barberellus*; cluster 4, all *An. obscurus* plus one picture of *An. nili* Theobald, 1904 and *An. paludis*; cluster 7 gathering all *An. arabiensis* but including six extra-specie pictures and cluster 14, all *An. gambiae* plus three pictures of *An. colluzzii*. The last category of clusters included most pictures of a species, but not all of them: cluster 5, mainly *An. paludis*; cluster 6, primarily *An. maculipennis*; cluster 9, mainly *An. cinctus*; cluster 10, mainly *An. squamosus*; cluster 13, mostly *An. coluzzi* and cluster 8, gathering the *An. multicolor* photos with three pictures of *An. cinereus*. Notably, no species out of the 20 under study scattered into more than two clusters.

Finally, the reliability of the DL model to accurately classify WIPs pictures of 19 *Anopheles* species was calculated, and results are presented in Table 4. Variable level of accuracy is recorded, ranging from faintly (50.00%) to perfect classification (100.00%). A perfect accuracy (100.00% level) is achieved for ten species whose WIPs pictures were filled in the dataset. More than 50% of accuracy in classification is recorded for three species, but the DL methods failed to assign 7 *Anopheles* species with an accuracy superior to 50% (Table 4). For most of the species whose assignation accuracy falls below 70%, a low number of representative pictures is available; indeed, only a small number of pictures are available for the test process (*An. demeilloni* 2, *An. maculipennis* 4, *An. barberelus*, 2, etc.). More than ten pictures per species might be a prerequisite to get good accuracy with our process; this will be further investigated. Only 14 pictures of the test dataset were misclassified (Fig. 2B), and the computed specific recognition of *Anopheles* remains astonishing, considering our dataset's species richness.

**Table 4:**
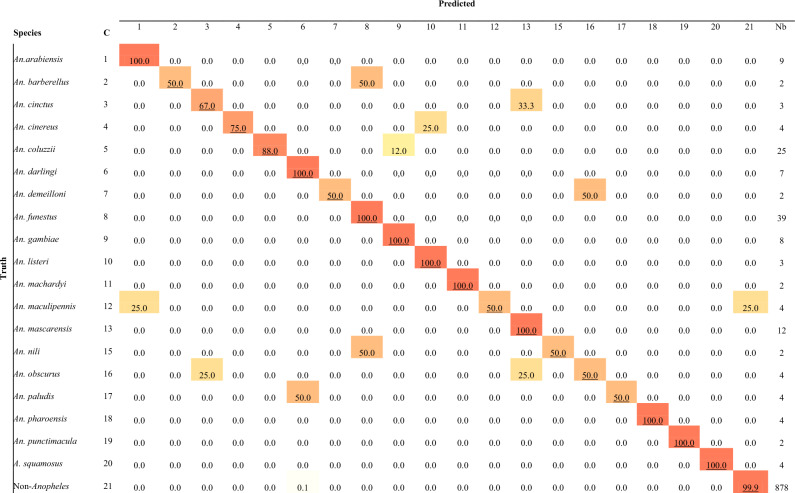
Accuracy tests of the deep learning at the species level.

*C* class number, *Nb* number of samples tested, *NA* not ascertained.

The *Anopheles* genus encompasses numerous morphologically indistinguishable species, ranging into the species complex level, e.g., ‘morphologically similar or identical natural populations that are reproductively isolated’. According to this definition, 27 species complexes are currently described for *Anopheles*. Our dataset gathers specimens from 4 complexes, the Claviger, Gambiae, Marshallii, and Nili complexes. *Anopheles nili* belongs to a complex of 4 species (*An. nili*; *An. somalicus* Rivola & Holstein, 1957; *An. carnevalei* Brunhes, Le Goff & Geoffroy, 1999; *An. ovengensis* Awono-Ambene, Kengne, Simard, Antonio-Nkondjio & Fontenille, 2004). Unfortunately, we cannot address the accuracy of the identification process for 3 of them because species were documented in our dataset with less than ten pictures for the Marshallii and Claviger complexes, or only one species for the Nili complex.

**Figure 5:**
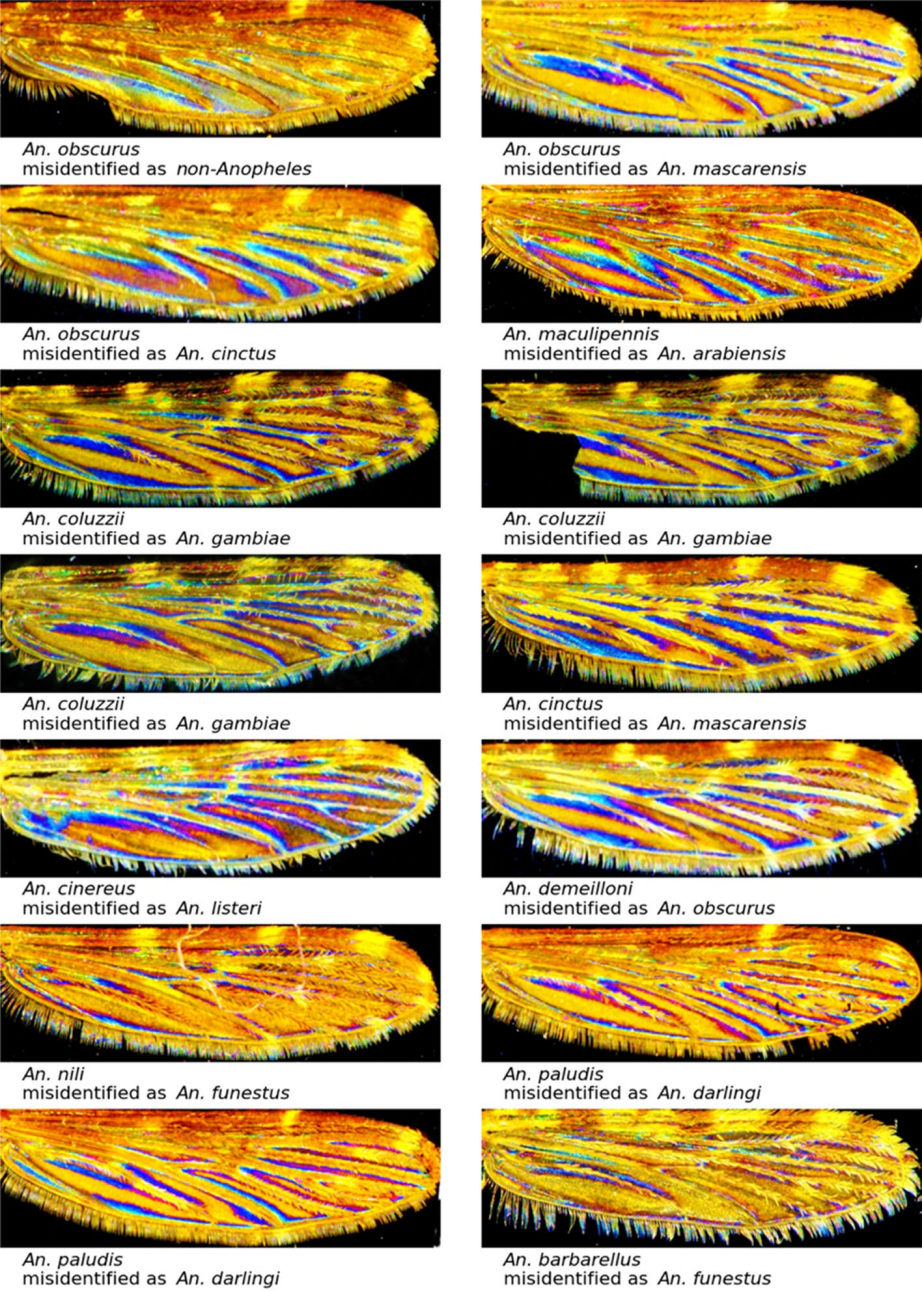
Selected examples of misclassified pictures at the genus level (*An. obscurus* misidentified as non-*Anopheles*), at the species level, and within the Gambiae complex.

As early as 1968, morphological variations in *An. nili s.l* populations suggest that *An. nili* is a complex of species whose members were further identified^44,45^. Our set of pictures of *An. nili,* gathers specimens date back to 1966 and might, therefore, encompass species belonging to the Nili complex not described before shreds of evidence for the presence of this complex. In addition to the few pictures available to train the classifier, other underlying factors might result in the fair identification accuracy we recorded. For other mispredicted pictures, the small number of samples available and the age of specimens age might have altered the prediction approach’s power. Even if *An. maculipennis* can be misidentified as *An. arabiensis* see Fig. 5, these two species are not sympatric in their natural environment.

The “Gambiae complex^9^”, first described in 1960, is documented in our dataset by four species over the nine currently described. Nevertheless, fewer than ten pictures are present in the dataset for one species. Nevertheless, our DL approach on WIPs demonstrates an astonishing identification accuracy of 100% for *An. arabiensis* and *An. gambiae* and 88% for *An. coluzzii* (Table 4). All specimens were collected from colonies avoiding misidentification ambiguity. Strikingly *An. coluzzii* is frequently misidentified as *An. gambiae* but never as *An. arabiensis*. It appears that *An. gambiae* and *An. arabiensis* are always correctly identified.

Overall photos of mispredicted species (Fig. 5) show that the samples of *An. obscurus* miss-predicted as a non-*Anopheles* specimen is of interest since this specimen bears wings characters of *Anopheles*, smooth and patchy areas on the wing costa and subcosta. This sample originated from Congo and was collected in 1988; the interferential pattern was still present but appeared slightly degraded during the preservation period. Such modification might have affected the recognition process, and it is documented that some slight picture modifications (blur lens, etc..) can significantly alter the recognition accuracy of our process^27^. The *An. coluzzii* misidentified as *An. gambiae* presents morphological alteration with damage on the wing; nevertheless, this hasn’t prevented a correct classification at the genus and subgenus taxonomic level.

## Discussion

In this study, we present clues on the accuracy of WIPs with DL to identify *Anopheles* specimens at various taxonomic levels, genus of subgenus, species, and complexes. Our results reveal that WIPs generated at the surface of *Anopheles* wings are a proper fingerprinting method to decipher specimens’ identity at taxonomic levels of interest for the entomological survey and vector control follow-up.

Since the 2010s, WIPs (Wing Interference Patterns) have received significant attention for their potential as a diagnostic method for species identification, used in taxonomic and systematic studies^23,25,46^. The transparent wings with a thin membrane*, i.e*., mainly in small insects, allow the formation of a colored pattern via thin-film interference. In a dark and light-absorbing environment with incoming external light (sunshine, for example), conspicuous WIPs are displayed on the wing membranes. These WIPs significantly vary among specimens belonging to distinct species but moderately between specimens of the same species or between sexes. The observed newton color series is similar to that appearing on a soap bubble and is directly proportional to the thickness of the wing membrane at any given point. Unlike the angle-dependent iridescence effect of a flat film, wing structures in an insect’s thin wing membrane act as diopters ensuring the WIPs appear essentially non-iridescent^23^. The role played by WIPs on sexual selection in *Drosophila melanogaster* was addressed, demonstrating that males with more vivid wings are more attractive to females than males with dull wings. These experimental results add a visual element to the mating tool array of *Drosophila*^47^. The role of WIP during courtship points to a function during insects’ speciation. This point is interesting and will deserve further exploration for explaining assortative mating of the Gambiae complex members in their natural environment.

The genus *Anopheles* encompasses eight subgenera, *Anopheles*, *Baimaia, Cellia, Christya*, *Kerteszia*, *Lophopodomyia*, *Nyssorhynchus,* and *Stethomyia*. The largest cosmopolitan genera are *Anopheles* and *Cellia*. From a malaria transmission standpoint, a relatively small number of species of the *Cellia* subgenus (i.e., the Gambiae complex) are responsible for most of the world’s malaria transmission; for a broader entomological survey point of view, more than 100 species of *Anopheles* are of medical and veterinary interest. With about 500 inventoried and validated species, accurately identifying *Anopheles* is challenging, even using published identification keys^48–52^. The presence of species complexes further puzzled the survey in areas where vector and non-vector species belonging to the same complex are sympatric. The two most known examples of such complexes are the *An. maculipennis* complex, with at least nine species in Europe^45^, and the Gambiae complex, with nine species in Africa. Besides diversity, microscopic observation is a time-consuming and challenging process, mainly owing to the skills and experience of dedicated public health personnel. In addition, variability in the morphological characteristics of mosquitoes collected in the fields may be degraded due to discoloration or damages caused during the capture and processing at the study site or during the freezing and drying preservation protocols.

Methodologies relying on genetic or biochemical criteria were tested to overcome such identification challenges. The DNA employs short molecular sequence tags from standardized genomic regions for species identification. Actually, 16,948 records forming 378 clusters, are available for the *Anopheles* genus (http://v4.boldsystems.org/index.php request performed on 12/7/2022). DNA barcoding can complement the morphological assessment of specimens but present several flattens and needs to be better suited for field entomological surveys^53,54^. Biochemical markers include protein profiling using MALDI-TOF analysis or other biochemical characteristics of the sample, like the cuticle carbohydrate composition and chemical formula^55^. The MALDI-TOF profiling was first applied with relative success to a restricted number of *Anopheles* species (*An. albimanus*, *An. minimus*, *An. freeborni*, *An. farauti*, *An. atroparvus, An. funestus*), but including members of the Gambiae complex (*An. quadrimaculatus*, *An. merus*, *An. gambiae*, *An. arabiensis*), using head and thoraces of females mosquitoes^56,57^. This methodology was further applied to some neotropical anophele vectors (*An. albimanus*, *An. apimacula*, *An. aquasalis*, *An. darlingi*, *An. malefactor*, *An. nuneztovari*, *An. pseudopunctipennis*, *An. punctimacula*) with an identification success between 78 to 100%, comparable to our accuracy rate^58^. Nevertheless, few works were subsequently performed on *Anopheles* specimens with protein profiling^59^. Altogether, these methodology helps to solve some taxonomic and ambiguous identification problems but could not be amenable for entomological survey purposes due to their cost, requirements in infrastructures and material, and trained personnel. Infrared spectroscopy (NIR and MIR) can detect changes in mosquito cuticles by quantifying light absorbed^60^. The discriminative capability of such methodology at the species level has yet to be thoroughly investigated and is restricted to very few members of the *Anopheles* genus, i.e., *An. gambiae s.s, An. coluzzii*, and *An. arabiensis* but appears to be well fitted for age grading of populations^34,60–65^, but also, interestingly, on pathogen (*Plasmodium*) detection within the arthropod vector^66^. Here, we provide clues on the reliable Anopheles species identification using WIPs and the DL process. We identified some species with 100% accuracy, even those belonging to the Gambiae complex of species^34,62^. This precision is higher than those provided by MIR or NIR technology. We also provide pieces of information on the capability of this method to be successfully translated on field-collected samples and old specimens. In addition, our methodology allows for identifying specimens at various taxonomic levels and, even for damaged specimens, addressing classification at the genus and/or subgenus levels. This is of interest for medical entomology purposes, knowing that species having a medical or veterinary interest are gathered in four out of the eight subgenera described. It might also be helpful for taxonomic studies involving old specimens.

The advance in Deep learning (DL) processes have opened a new perspective for arthropod identification. This branch of machine learning has the versatility to be employed on various markers of use in entomology, including protein profiling and image analysis for morphological characteristics. The latter, which includes typical morphological characteristics used to identify Culicidae specimens, can potentially be used in “citizen sciences” projects. Such community surveillance has been applied for mosquitoes ^67,68^, and a citizen science approach in conjunction with a deep learning method was developed to follow *Aedes albopictus* (Skuse, 1895) from pictures taken by citizen^69^. Nevertheless, for instance, the accuracy of such methods has not been evaluated in areas with high Culicidae diversity or for *Anopheles* recognition. Nonetheless, we can anticipate that this process will suffer from the same limitation in identifying species belonging to the same complex. Although DL approaches have also been applied for training *Anopheles* belonging to the Gambiae complex identification and age grading using MIR^34^, its interest for a taxonomic purpose has not been thoroughly probed.

Therefore, it will be of interest to further explore the capacity of WIPs in couple with DL to address challenges concerning delineating geographically distinct populations, sex, physiological state identification, and its versatility to perform age grading in natural populations, if any. We anticipate this method can be applied to other arthropod vector-borne diseases. Assuming that Deep learning analysis results in robust classification outcomes, it is worth evaluating, even qualitatively, whether the proposed approach could be scalable and usable in real-life conditions regarding several essential criteria: cost (infrastructure, material, technically skilled personnel), computational resources, analyzing time, sample destructiveness, and taxonomic level of the classification.

## Data Availability

The source code is publicly available on GitHub, with a direct https://github.com/marcensea/diptera-wips.git. Dataset is available with a direct https://doi.org/10.6084/m9.figshare.22083050.v1.
